# Nanoporous Gold-Modified Screen-Printed Electrodes for the Simultaneous Determination of Pb^2+^ and Cu^2+^ in Water

**DOI:** 10.3390/s24175745

**Published:** 2024-09-04

**Authors:** Yongfang Li, Xuan Chen, Zhiyong Yuan, Zhijian Yi, Zijun Wang, Rui Wang

**Affiliations:** 1 School of Food Science and Engineering, Foshan University, Foshan 528231, China; 18110220111@fudan.edu.cn (Y.L.); 2112358077@stu.fosu.edu.cn (X.C.); 20220400221@stu.fosu.edu.cn (Z.Y.); 2111958057@stu.fosu.edu.cn (Z.Y.); 20200400201@stu.fosu.edu.cn (Z.W.); 2 Human Phenome Institute, State Key Laboratory of Genetic Engineering, Fudan University, Shanghai 200438, China; 3 Center for Medical Research and Innovation, Shanghai Pudong Hospital, Fudan University Pudong Medical Center, Shanghai 200438, China; 4 International Human Phenome Institutes, Shanghai 200438, China

**Keywords:** nanoporous gold, screen-printed carbon electrode, dynamic hydrogen bubble template, electrochemical sensor, heavy metal

## Abstract

In this study, nanoporous gold (NPG) was deposited on a screen-printed carbon electrode (SPCE) by the dynamic hydrogen bubble template (DHBT) method to prepare an electrochemical sensor for the simultaneous determination of Pb^2+^ and Cu^2+^ by square wave anodic stripping voltammetry (SWASV). The electrodeposition potential and electrodeposition time for NPG/SPCE preparation were investigated thoroughly. Scanning electron microscopy (SEM) and energy-dispersive X-ray diffraction (EDX) analysis confirmed successful fabrication of the NPG-modified electrode. Electrochemical characterization exhibits its superior electron transfer ability compared with bare and nanogold-modified electrodes. After a comprehensive optimization, Pb^2+^ and Cu^2+^ were simultaneously determined with linear range of 1–100 μg/L for Pb^2+^ and 10–100 μg/L for Cu^2+^, respectively. The limits of detection were determined to be 0.4 μg/L and 5.4 μg/L for Pb^2+^ and Cu^2+^, respectively. This method offers a broad linear detection range, a low detection limit, and good reliability for heavy metal determination in drinking water. These results suggest that NPG/SPCE holds great promise in environmental and food applications.

## 1. Introduction

Heavy metals are a group of metallic elements with atomic densities exceeding 5 g/m^3^, encompassing mercury, lead, cadmium, copper, and arsenic [1,2]. With the rapid pace of global industrialization and urbanization, heavy metal pollution has emerged as an increasingly prominent concern. Upon absorbed into the human body, lead (Pb) circulates through the bloodstream and accumulates in vital organs, leading to detrimental effects on the liver, kidney, and nervous system [3]. While Cu^2+^ is a necessary metallic element for the human body, excessive intake can also bring various issues on the central nervous system, gastrointestinal system, and kidney, damaging body health [4]. Therefore, it is necessary to develop sensitive and efficient detection techniques for the monitoring of Pb^2+^ and Cu^2+^ in environmental and food samples, thus defending heavy metal pollution and ensuring food safety.

For rapid determination of metal ions, chemosensors that utilize the fluorescence variation before and after the binding with ions were preferred due to their non-destructive measurement and rapid analysis. However, their high background interference may impair their sensitivity [5]. Electrochemical methods, especially electrochemical stripping analysis, have been widely recognized as powerful tools for determination of heavy metals [6]. In this method, heavy metal ions enrich on the electrode under a constant potential, then strip out for detection. Each metal ion has its own characteristic stripping potential, and the generated current is proportional to the amount of metal ion [7,8]. Heavy metal ions with different stripping potentials can be determined simultaneously. Electrochemical stripping analysis has the advantages of being cost-effective, easy-handling, high sensitivity, and rapidness [9,10]. Working electrode modification is important in the construction of electrochemical sensors since it can enhance the active area of the electrode surface and improve the electron transfer rate [11].

Nanogold is a good choice for the modification of electrodes due to its high conductivity and high affinity for some heavy metals. It has been widely used in the heavy metal determination [12,13]. While nanoporous gold (NPG) is a three-dimensional porous material with a high specific surface area [14,15]. Modifying electrodes with NPG can further improve their electrocatalytic performance, increase the active surface area, and provide more active sites for reactions [16]. Therefore, nanoporous gold holds broad prospects for heavy metal detection. The preparation methods of nanoporous gold mainly include dealloying, the template method, and anodization [17]. These methods are difficult to apply to screen-printed electrodes due to the use of concentrated acids and the complexity of template removal. The dynamic hydrogen bubble template (DHBT) method is an effective electrodeposition technique for preparing three-dimensional porous materials [18]. In this method, the generation of hydrogen bubbles and the deposition of metal ions are conducted simultaneously under a high density of cathodic current to create porous nanostructures. This method does not require inorganic or organic materials as templates. Moreover, the modification can be achieved merely by a potentiostat, which is facile and convenient [19].

**Scheme 1:**
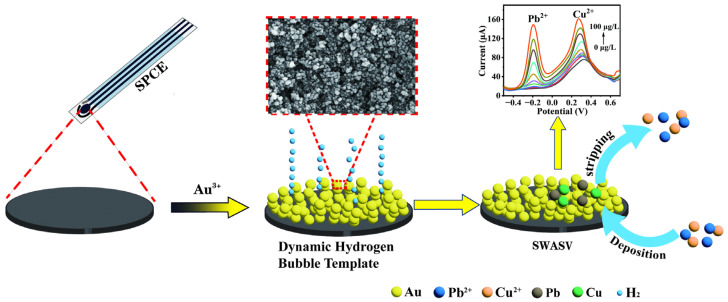
Electrodeposition of nanoporous gold on screen-printed carbon electrodes by the hydrogen bubble templating method for simultaneous determination of Pb^2+^ and Cu^2+^ through SWASV.

In this study, nanoporous gold was electrodeposited on a screen-printed carbon electrode (SPCE) by the dynamic hydrogen bubble template method, thus preparing a nanoporous gold modified screen-printed electrode (NPG/SPCE). Then, Pb^2+^ and Cu^2+^ were simultaneously determined through square wave anodic stripping voltammetry (SWASV), where Pb^2+^ and Cu^2+^ were deposited on the NPG/SPCE and then stripped out to the solution with two pronounced and independent stripping peaks (Scheme 1). The electrodeposition potential and electrodeposition time for the preparation of NPG/SPCEs were systematically investigated. For comparison, conventionally nanogold-modified SPCE (AuNPs/SPCE) was also prepared. With NPG/SPCE, Pb^2+^ and Cu^2+^ were simultaneously determined with wide linear range, low limits of detection, and good repeatability and specificity. Furthermore, the analysis results of water samples were consistent with those obtained from the ICP-MS method, validating the potential application of this NPG/SPCE-based sensor.

## 2. Materials and Methods

### 2.1. Reagents and Materials

Chloroauric acid, potassium chloride, potassium ferrocyanide trihydrate, and potassium ferricyanide were purchased from Shanghai Macklin Biochemical Technology Co., Ltd. (Shanghai, China). Stock solutions of Pb^2+^ (1000 μg/mL) and Cu^2+^ (1000 μg/mL) were purchased from Beijing General Research Institute for Nonferrous Metals. Hydrochloric acid, sulfuric acid, nitric acid, anhydrous disodium hydrogen phosphate, disodium hydrogen phosphate, acetic acid, calcium nitrate, magnesium nitrate, potassium nitrate, and sodium nitrate were purchased from Guangzhou Chemical Reagent Factory, Guangzhou, China. The screen-printed carbon electrode (diameter 2.8 mm) was purchased from Wuhan Zhongkezhikang Biotechnology Co., Ltd. (Wuhan, China). Microporous filter membrane (0.22 μm) was purchased from Bikeman Biotechnology Co., Ltd. (USA). All solutions were prepared using ultrapure water produced by Milli-Q ultrapure water (Millipore Company, Danvers, MA, USA).

### 2.2. Instruments

Scanning electron microscope (SEM) images were acquired on a field emission scanning electron microscope (Quattro S, Thermo Fisher Scientific, Waltham, MA, USA USA). Energy dispersive X-ray (EDX) spectrum was collected on an energy dispersive spectrometer (Ultim Max Oxford Instruments Ltd., Oxford, UK). Electrochemical impedance spectroscopy (EIS) was performed with a potentiostat (Autolab 302N, Metrohm Autolab B.V., Herisau, Switzerland). Other electrochemical experiments were performed on a potentiostat (CHI1440 Shanghai Chenhua Instrument Co., Ltd., Shanghai, China). A SPCE connector and a time- and speed-regulated stirring device were self-made for heavy metal detection.

### 2.3. Experimental Methods

#### 2.3.1. Electrode Modification

The screen-printed carbon electrode used in our study consisted of a carbon working electrode, a carbon counter electrode, and a Ag/AgCl reference electrode. The SPCE was washed with ultra-pure water and ethanol three times alternatively to remove the impurities adhering to the electrode surface. Then, it was dried naturally at room temperature. A total of 4 mM HAuCl_4_ solution containing 0.5 M H_2_SO_4_ was dropped on the electrode surface. Then, nanogold or nanoporous gold was electrodeposited on the SPCE under a fixed applied potential. The deposition potential was set as −0.6 V and −3.0 V for the modification of nanogold and nanoporous gold, respectively. The deposition time was set at 40 s. After modification, the SPCE was rinsed with ultra-pure water to remove the residual HAuCl_4_ and H_2_SO_4_.

#### 2.3.2. SWASV Determination of Pb^2+^ and Cu^2+^

A total of 1 mL of 0.1 mol/L HCl solution was used as the supporting electrolyte, and a certain amount of Pb^2+^ and Cu^2+^ were added. The modified electrode was rinsed in the electrolyte, and then Pb^2+^ and Cu^2+^ were detected by SWASV. The parameters were set as follows: The deposition potential was −0.5 V, the deposition time was 300 s, the potential increment was 4 mV, and the frequency was 25 Hz. In the deposition process, a time- and speed-regulated stirring device was used to rotate the sample cell, and the stirring rate was 200 rpm. No stirring was provided in the stripping process. All tests were performed at room temperature.

### 2.4. Real Sample Analysis

Bottled drinking water and tap water samples absent of Pb^2+^ and Cu^2+^ were used for spiking analysis. The water samples were filtered by 0.22 μm microporous filter membrane, followed by the addition of an equal volume of 0.2 M HCl. After thorough mixing, a specific amount of Pb^2+^ and Cu^2+^ were added to the sample solution to obtain spiked samples with different concentrations of Pb^2+^ and Cu^2+^. The spiked samples were subjected to electrochemical detection by the method described in Section 2.3.2. For evaluation of the constructed electrochemical sensor’s reliability toward Pb^2+^ and Cu^2+^, the result of electrochemical analysis is compared with that of ICP-MS.

## 3. Results and Discussion

### 3.1. Investigation of NPG/SPCE Preparation

In this part, the hydrogen evolution potential and the reduction potential of AuCl_4_^−^ were studied through the cyclic voltammetry characteristics of SPCEs in deposition solution. Then, the effects of deposition potential and deposition time on the preparation of NPG/SPCEs was investigated.

#### 3.1.1. Study on Cyclic Voltammetry Characteristics of SPCE in Deposition Solution

The electrochemical behavior of SPCE in the deposition solution (4 mM HAuCl_4_ containing 0.5 M H_2_SO_4_) was studied by cyclic voltammetry (CV). The scanning range was 1.4~−0.35 V, and the scanning rate was 0.1 V/s. Two scanning cycles were conducted.

**Figure 1:**
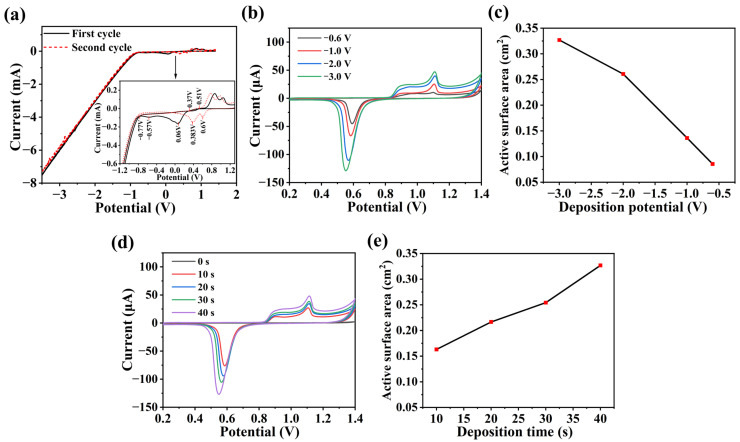
Investigation of electrodeposition conditions for NPG/SPCE preparation. (**a**) Cyclic voltammetry curve of screen-printed electrode in deposition solution (4 mM HAuCl_4_ containing 0.5 M H_2_SO_4_). (**b**) CV curves in 0.5 M H_2_SO_4_ and (**c**) calculated active surface areas of SPCEs prepared at different deposition potentials. (**d**) CV curves in 0.5 M H_2_SO_4_ and (**e**) calculated active surface areas of NPG-SPCEs prepared at different deposition times.

As shown in Figure 1a, in the former segment of the first scanning cycle (solid line), there is no cathodic current when the potential is higher than 0.52 V, indicating that Au was not generated. As the potential decreases, cathodic current significantly increases, and a large cathodic current peak appears at 0.06 V, indicating that Au^3+^ was reduced to Au, and the Au nucleates and grows on the SPCE surface substantially [20]. The reduction process is shown in Equation (1):

[AuCl_4_]^−^ + 3e^−^ → Au + 4Cl (1)

When further scanning towards the negative potential, a small current peak appears at −0.57 V. When the potential is lower than −0.77 V, the current increases sharply, which is caused by the evolution of a large amount of hydrogen from the surface of the working electrode. The reduction process is shown in Equation (2):

2H^+^ + 2e^−^ → H_2_ (2)

When the latter segment of the first scanning cycle was conducted, a current crossover occurred at 0.37 V. This potential is the overpotential for nucleation. At this potential, nucleation and growth proceed at a measurable rate [21]. Another current crossover occurs at 0.51 V, where the equilibrium of Au^3+^/Au redox reaction is achieved. Anodic current and anodic current peaks appeared at the potential range of 0.51~1.4 V, indicating the oxidation of Au. In the second scanning cycle (dashed line), the larger cathodic current peak shifts from 0.06 V to 0.383 V, and a small cathodic current peak appears at 0.60 V, indicating that within the potential range of 0.06~0.383 V, Au (0) is more easily deposited on the Au generated in the first scanning cycle [22]. By studying the cyclic voltammetry curve of the screen-printed electrode in the deposition solution, the variation of Au^3+^/Au under different potentials can be cleared, which offers theoretical support for the electrochemical deposition of gold materials on SPCEs.

#### 3.1.2. Optimization of Deposition Potential and Deposition Time

Nanogold can be electrodeposited on the electrode under a certain deposition potential and deposition time, where Au^3+^ in the solution is reduced to Au and modified on the electrode. The applied deposition potential is commonly non-hydrogen evolution potential. While nanoporous gold with high specific surface area can be obtained by utilizing the hydrogen evolution potential, known as the hydrogen bubble template method [23,24]. The size and the rate of evolution of hydrogen bubbles can provide a dynamic template for depositing metal atoms, thus generating porous materials with 3D nanoarchitectures. It is reported that deposition potential and deposition time strongly influence the porosity, grain size, and density of the nanofilms [25]. Therefore, the electrochemical characterizations and the sensing effect can be altered by optimizing the electrodeposition parameters. Herein, we use the active surface area to identify the optimal deposition potential and deposition time for NPG/SPCE preparation. The active surface area of modified SPCE was measured by CV test, which is conducted in 0.5 M H_2_SO_4_ with a scanning range of 0.2~1.4 V and a scanning rate of 0.1 V/s. The reduction peak of gold oxide can be observed in the CV curves. The active surface area of the NPG–electrode can be calculated by Equations (3) and (4) [26,27]:

Q_mes_ = S/*v* (3)

R_sa_ = Q_mes_/Q_sp_ (4)

where Q_mes_ is the total charge of reduction process (C); S is the integrated area of reduction peak (CV/s); *v* is the scanning rate (V/s); R_sa_ is the active surface area (cm^2^); and Q_sp_ is the theoretical charge density for gold oxide reduction (390 μC cm^−2^) [28,29].

Firstly, the deposition potential for the preparation of nanoporous gold was investigated. The electrodeposition was conducted under hydrogen evolution potential and non-hydrogen evolution potential, respectively. According to Figure 1a, the hydrogen evolution occurs under a potential lower than −0.77 V. Therefore, −0.6 V is selected as the non-hydrogen evolution potential for deposition, −1.0 V, −2.0 V, and −3.0 V are selected as the hydrogen evolution potential.

During the deposition process, it can be observed that when the deposition potential is −0.6 V, there is no evolution of hydrogen on the electrode surface. When the deposition potential is −1.0 V, significant hydrogen evolution occurs on the electrode surface, with hydrogen bubbles slowly growing larger and adhering to the working electrode surface. Compared to −0.6 V, the deposition rate became higher, and for that, the electrode quickly turned yellow. However, oversized hydrogen bubbles hinder the deposition of gold, resulting in noticeable vacancies on the modified electrode surface [30]. As the deposition potential continues to move towards −3.0 V, dense hydrogen bubbles generate on the electrode surface, which can prevent the enlargement and adhesion of hydrogen bubbles, thus facilitating the generation of nanoporous gold [31].

The CV curves of electrodes prepared at different electrodeposition potentials are shown in Figure 1b. Compared to the electrode prepared under the non-hydrogen evolution potential (−0.6 V), the NPG-modified electrodes using the hydrogen evolution potential possess a higher reduction peak current. In addition, as the deposition potential decreases, the peak current significantly increases and reaches a maximum at −3.0 V, at which point the active surface area (0.327 cm^2^) is approximately 3.85 times that of the electrode deposited at −0.6 V (0.085 cm^2^) (Figure 1c). This indicates that the nanoporous gold-modified SPCE has a larger active surface area than conventional nanogold-modified electrodes. Further lowering the deposition potential will result in excessive current during deposition, which may exceed the range of the potentiostat (±10 mA) and damage the instrument. Taken together, a deposition potential of −3.0 V is selected for the electrodeposition of NPG in subsequent experiments.

The deposition time for preparing NPG was also investigated. The deposition potential was set as −3.0 V, and the deposition time ranged from 0 s to 40 s. As shown in Figure 1d, there is no reduction peak of gold oxide in the CV curve for the deposition time of 0 s, indicating that nanoporous gold was not electrodeposited on the electrode surface. In addition, the reduction peak current of gold oxide increases as the deposition time ranges from 10 s to 40 s, indicating the increased deposition amount of NPG on the electrode surface with electrodeposition time rising up. Moreover, the calculated active surface area of the NPG-modified electrode prepared at 40 s (0.327 cm^2^) is approximately two times that at 10 s (0.163 cm^2^). Considering the modification efficiency and the stability of the baseline during subsequent stripping detection, 40 s is chosen as the deposition time for the preparation of nanoporous gold.

### 3.2. Morphological Characterization and Element Analysis of SPCE

**Figure 2:**
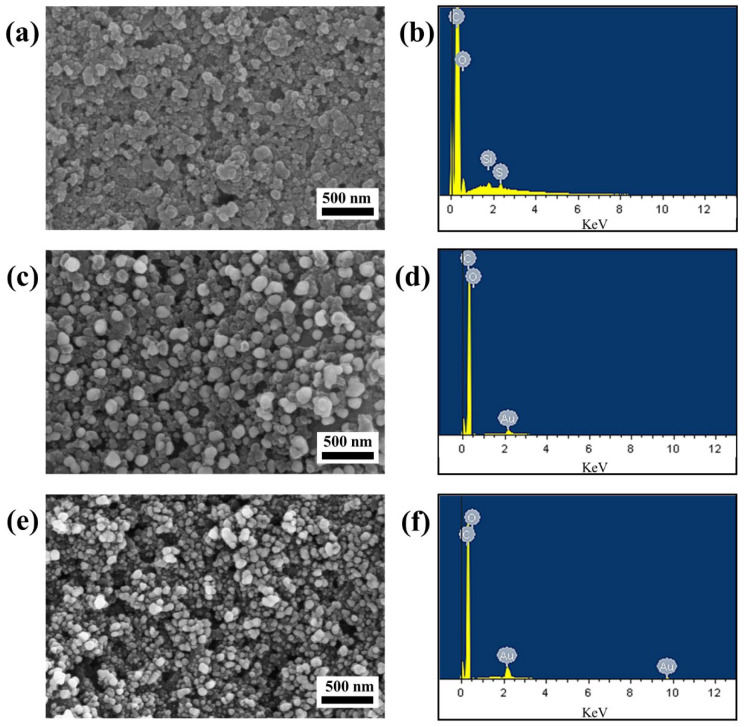
SEM images of bare SPCE (**a**), AuNPs/SPCE, (**c**) and NPG/SPCE (**e**). EDX spectrums of bare SPCE (**b**), AuNPs/SPCE, (**d**) and NPG/SPCE (**f**).

SEM and EDX were used to characterize the surface of SPCE modified with nanogold (AuNPs/SPCE) and nanoporous gold (NPG/SPCE). It can be observed that there are great changes in the morphology of electrodes after electrodeposition (Figure 2a,c,e). Nanoparticles of different sizes were modified on the electrode. The changes in EDX spectrums demonstrated that the modified material is gold (Figure 2b,d,f). The carbon, oxygen, and silicon were from the bare screen-printed carbon electrode (Figure 2b). The average particle size of AuNPs electrodeposited at −0.6 V was approximately 102 nm (Figure 2c), whereas the average particle size of AuNPs electrodeposited at −3.0 V was about 60 nm (Figure 2e). These gold nanoparticles aggregated during the deposition process and formed a loose porous structure, demonstrating the successful preparation of NPG/SPCE.

### 3.3. Electrochemical Characterization of NPG/SPCE

#### 3.3.1. Electrode Evaluation with Cyclic Voltammetry

**Figure 3:**
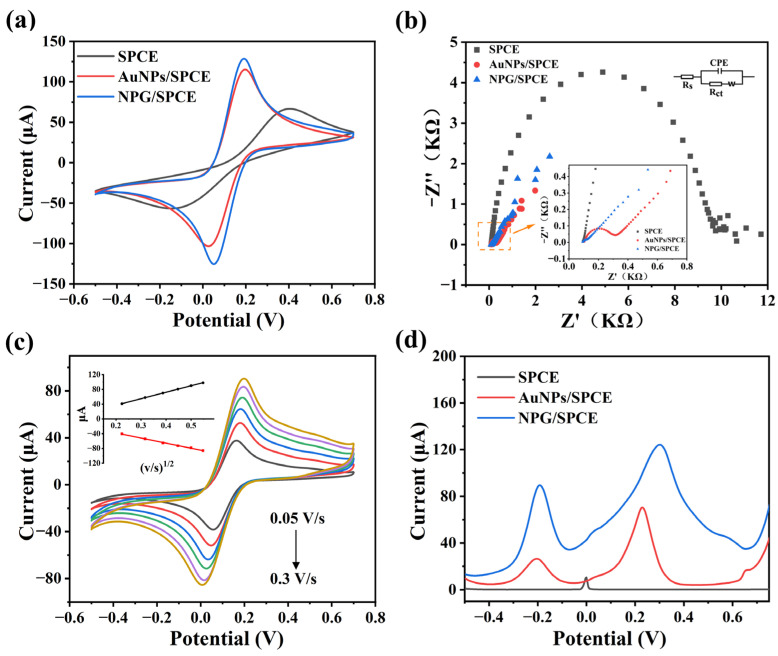
Electrochemical characterization of NPG/SPCE. Cyclic voltammetry curves (**a**) and electrochemical impedance spectroscopy curves (**b**) of bare electrode, AuNPs/SPCE, and NPG/SPCE in 5 mmol/L [Fe (CN)_6_]^3−/4−^ and 0.1 mol/L KCl solution. (**c**) Cyclic voltammetry curves of NPG/SPCE in 5 mmol/L [Fe (CN)_6_]^3−/4−^ and 0.1 mol/L KCl solution at different scan rates. Inset is the linear relationship between redox peak current and the square root of scan rate; (**d**) square wave stripping voltammetry curves of Pb^2+^ and Cu^2+^ detected by different electrodes.

To evaluate the conductivity of NPG/SPCE, CV scans were performed using NPG/SPCE, AuNPs/SPCE, and a bare electrode in a mixed solution of 5 mmol/L [Fe (CN)_6_]^3−/4−^ and 0.1 mol/L KCl, respectively. The scanning range was −0.5~0.7 V, and the scanning rate was 0.1 V/s. As shown in Figure 3a, the redox current using AuNPs/SPCE was much higher than that of the bare electrode, and the peak potential difference narrowed. This is because the modified gold nanoparticles enlarged the active surface area of electrode and improved the electron transfer ability [8]. In addition, the redox current further increased when using NPG/SPCEs, indicating that nanoporous gold nanoparticles further enlarged the active surface area of the electrode due to its 3D nano-architectures, thus providing more active sites for the redox reaction [16]. The EIS result was consistent with the CV test for that the Rct decreased with the modification of AuNPs and NPG, indicating enhanced electron transfer ability with different modifications (Figure 3b). The NPG/SPCE has a better electron transfer ability than the conventionally gold nanoparticle-modified electrode, demonstrating the great advantages of the 3D porous structure of nanoparticles for the electrode modification [30]. Then, CV tests using NPG/SPCEs under different scan rates were conducted. The redox current was linearly correlated with the square root of scanning rate with linear equations of Ipa = 175.57*v*^1/2^ + 2.10 (R^2^ = 0.9998) for the oxidation reaction and Ipc = −138.50*v*^1/2^ − 10.14 (R^2^ = 0.9974) for the reduction reaction (Figure 3c), indicating that the redox process of [Fe (CN)_6_]^3−/4−^ on the NPG/SPCE was mainly based on the linear diffusion control [32].

#### 3.3.2. Comparison of Electrode Performance for Heavy Metal Detection

To evaluate the real detection effect of different electrodes for the determination of Pb^2+^ and Cu^2+^, the SWASV test was carried out using bare electrodes, AuNPs/SPCE, and NPG/SPCE. As shown in Figure 3d, when using the bare electrode, only a small stripping peak of silver was observed, which is related to the reference electrode material [33]. When using AuNPs/SPCE, two independent stripping peaks of Pb^2+^ and Cu^2+^ appeared at −0.2 V and 0.23 V, respectively. As for the NPG/SPCE, the stripping peak of Pb^2+^ and Cu^2+^ appeared at −0.19 V and 0.3 V, respectively. Moreover, compared to the conventionally deposited AuNPs/SPCE, NPG/SPCE prepared by the dynamic hydrogen bubble template method obtained a higher peak current for Pb^2+^ and Cu^2+^ determination, which is due to the superior electron transfer ability of NPG/SPCE. Although the baseline was not smooth, it did not affect the detection. Therefore, NPG/SPCE demonstrated the best performance for the determination of Pb^2+^ and Cu^2+^.

### 3.4. Optimization of Experimental Parameters in SWASV

In order to improve the sensitivity of NPG/SPCE for the determination of Pb^2+^ and Cu^2+^, the supporting electrolyte, deposition potential, deposition time, and stirring rate used in the SWASV test were optimized.

#### 3.4.1. Supporting Electrolyte

**Figure 4:**
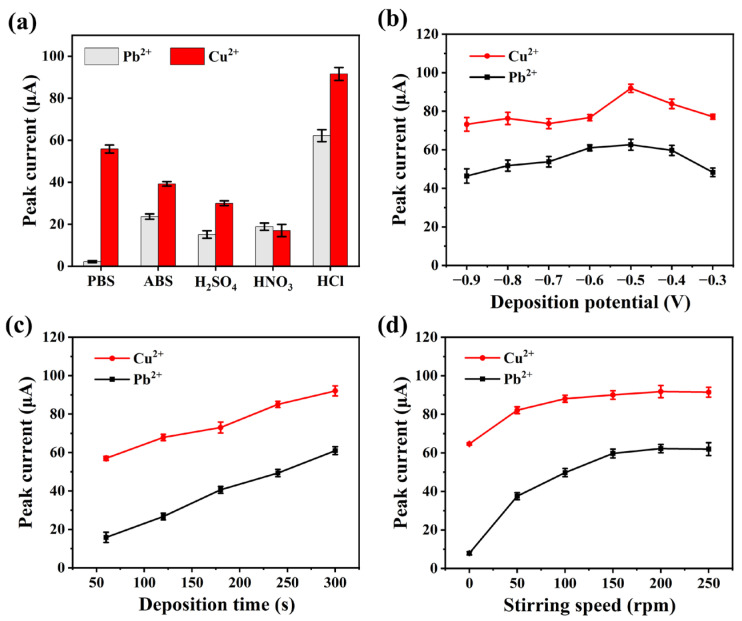
Optimization of experimental parameters for SWASV determination of Pb^2+^ and Cu^2+^. (**a**) Supporting electrolyte. (**b**) Deposition potential. (**c**) Deposition time. (**d**) Stirring speed.

Supporting electrolyte was tested among 0.1 M phosphate buffer solution (PBS, pH = 7), acetate buffer solution (ABS, pH = 4.5), H_2_SO_4_, HNO_3_, and HCl. Pb^2+^ and Cu^2+^ were added into the supporting electrolyte with a final concentration of 50 μg/L, respectively, then detected using NPG/SPCE through the SWASV method. As shown in Figure 4a, the highest stripping peak currents of Pb^2+^ and Cu^2+^ were observed when 0.1 M HCl solution was used as the supporting electrolyte. HCl is the commonly used supporting electrolyte in electrochemical analysis of heavy metals; for that, the chloride in the medium can improve the electrolytic conductivity. In addition, HCl can provide a suitable pH for the reaction to avoid the hydrolysis of heavy metals [34]. Therefore, a 0.1 M HCl solution was selected in subsequent experiments.

#### 3.4.2. Deposition Potential and Deposition Time

The deposition potential and deposition time in the enrichment process of SWASV have a significant impact on the detection of Pb^2+^ and Cu^2+^. Firstly, the influence of the deposition potential was investigated, which was set at −0.3, −0.4, −0.5, −0.6, −0.7, −0.8, and −0.9 V. As shown in Figure 4b, the stripping peak currents of Pb^2+^ and Cu^2+^ gradually increased as the deposition potential shifted from −0.3 V to −0.5 V, and reached the maximum at −0.5 V. When the deposition potential continued to shift towards −0.9 V, the peak currents of Pb^2+^ and Cu^2+^ decreased. This is because an excessively negative deposition potential can lead to a large extent of hydrogen evolution on the electrode surface during the enrichment process, hindering the enrichment of Pb^2+^ and Cu^2+^ and affecting subsequent stripping [35]. Therefore, −0.5 V was selected as the deposition potential for SWASV.

Next, the deposition time was investigated using the optimized conditions mentioned above, and the deposition time was set as 60, 120, 180, 240, and 300 s. As shown in Figure 4c, the peak currents of Pb^2+^ and Cu^2+^ increased linearly with the deposition time in the range of 60 to 300 s, suggesting that a longer deposition time is favored for the enrichment of Pb^2+^ and Cu^2+^ on the electrode surface. However, an excessively long deposition time will reduce the detection efficiency. Taken together, 300 s was adopted as the deposition time.

#### 3.4.3. Stirring Rate

Stirring during the enrichment process can reduce the concentration polarization, thus facilitating the enrichment of heavy metal ions. The stirring rate was adjusted by a self-made miniaturized device and set as 0, 50, 100, 150, 200, and 250 rpm. As shown in Figure 4d, the stripping peak currents of Pb^2+^ and Cu^2+^ significantly increased with the stirring rates ranging from 0 to 200 rpm and kept relatively stable after the speed exceeded 200 rpm. Therefore, 200 rpm was applied during the enrichment process.

### 3.5. Analytical Performance of NPG/SPCE for the Detection of Pb^2+^ and Cu^2+^

**Figure 5:**
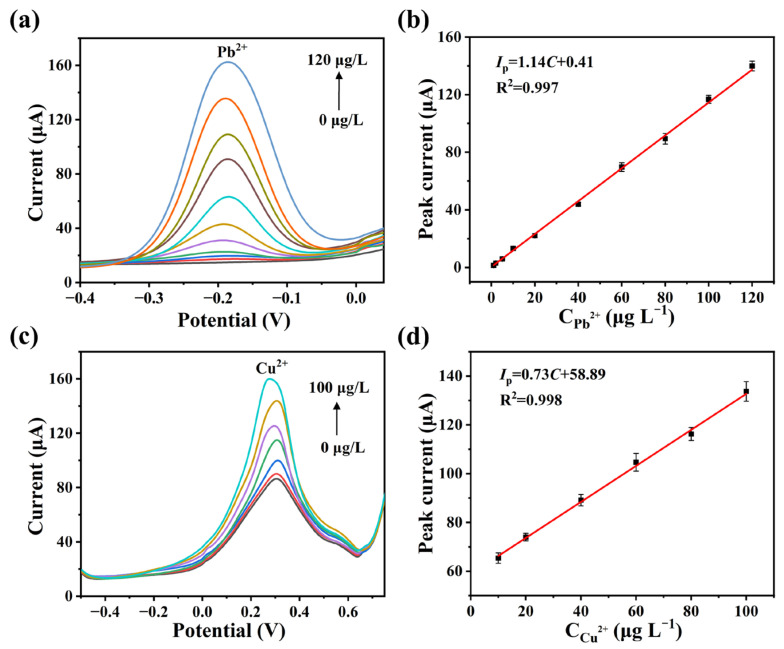
Analytical performance of NPG/SPCE for the individual determination of Pb^2+^ and Cu^2+^. SWASV curves (**a**) and standard curve (**b**) for the separate detection of Pb^2+^ with different concentrations. SWASV curves (**c**) and standard curve (**d**) for the separate detection of Cu^2+^ with different concentrations.

Under optimal experimental conditions, individual detection of Pb^2+^ using NPG/SPCE was conducted. The concentrations of Pb^2+^ were set as 0, 1, 2, 5, 10, 20, 40, 60, 80, 100, and 120 μg/L. As shown in Figure 5a, the stripping peak of Pb^2+^ appears at −0.19 V, and the stripping peak current increases with the Pb^2+^ concentration varying from 1 to 120 μg/L. The standard curve for Pb^2+^ determination is shown in Figure 5b. The linear regression equation is Ip = 1.14 C + 0.41, and R^2^ achieves 0.997. The limit of detection was calculated to be 0.4 μg/L (S/N = 3). The electrode exhibited good repeatability in the 10 consecutive detections of 50 μg/L Pb^2+^, with a relative standard deviation (RSD) of 3.00% (Figure S1a).

Subsequently, individual detection of Cu^2+^ at concentrations of 0, 10, 20, 40, 60, 80, and 100 μg/L was conducted with NPG/SPCE. As shown in Figure 5c, the stripping potential of Cu^2+^ is near 0.3 V. The stripping peak current is proportional to Cu^2+^ concentration within the range of 10–100 μg/L. The linear regression equation is Ip = 0.73C + 58.89, and R^2^ reaches 0.998 (Figure 5d). The limit of detection was determined to be 5.5 μg/L (S/N = 3). The electrode exhibited good repeatability in the 10 consecutive detections of 50 μg/L Cu^2+^, with an RSD of 2.61% (Figure S1b).

**Figure 6:**
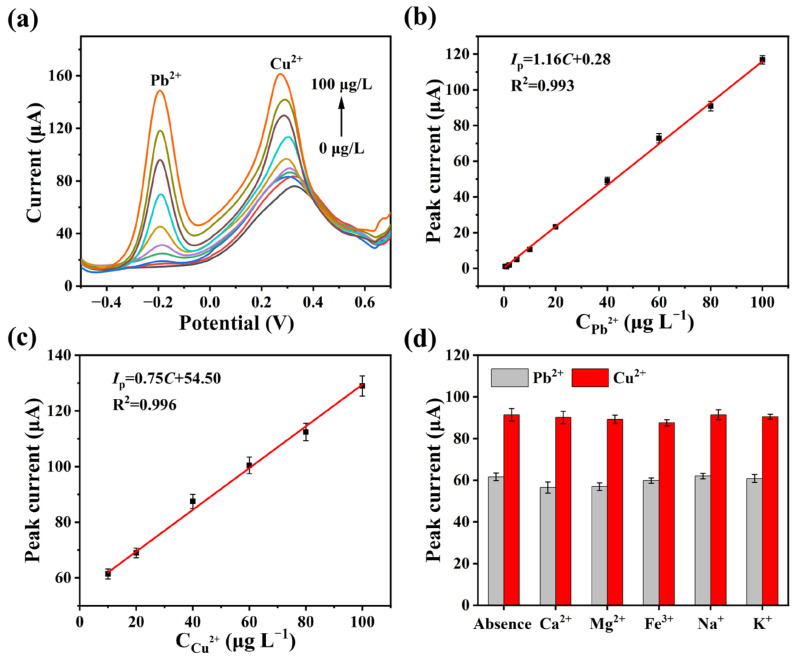
(**a**) SWASV curves for the simultaneous determination of Pb^2+^ and Cu^2+^ with different concentrations. Standard curve of Pb^2+^ (**b**) and Cu^2+^ (**c**) in a simultaneous determination. (**d**) Specificity test of NPG/SPCE. The interfering ion was added to the solution containing Pb^2+^ and Cu^2+^, and the corresponding peak current values of Pb^2+^ and Cu^2+^ were compared with those in a solution only containing Pb^2+^ and Cu^2+^ without any different ions (marked as absent).

Furthermore, simultaneous detection of Pb^2+^ and Cu^2+^ was performed using NPG/SPCE at concentrations ranging from 0 to 100 μg/L. As shown in Figure 6a, there are two distinct stripping peaks at −0.2 V and 0.3 V, which correspond to Pb^2+^ and Cu^2+^, respectively. In addition, the stripping peak currents present a good linear relationship with Pb^2+^ concentration within the range of 1–100 μg/L (Figure 6b). The linear regression equation is Ip = 1.16C + 0.28 (R^2^ = 0.993) and the limit of detection is 0.4 μg/L (S/N = 3). Similarly, as shown in Figure 6a,c, a good linear relationship is observed between stripping peak current and Cu^2+^ concentration within the range of 10–100 μg/L. The linear regression equation is Ip = 0.75C + 54.50 (R^2^ = 0.996), and the limit of detection is 5.4 μg/L (S/N = 3). Compared with the individual detection of Pb^2+^ and Cu^2+^, there are negligible changes in the slope of the linear regression equation and the limit of detection, indicating that the NPG/SPCE can achieve simultaneous detection of Pb^2+^ and Cu^2+^ without mutual influence. However, it was found that the linear range of Pb^2+^ decreased from 1–120 μg/L to 1–100 μg/L during simultaneous detection. This is due to the high total content of Pb^2+^ and Cu^2+^ in the test solution, leading to competitive binding of Pb^2+^ and Cu^2+^ at the working electrode surface [32]. The repeatability of NPG/SPCE in detecting Pb^2+^ and Cu^2+^ was good, with RSD values of 2.33% for Pb^2+^ and 2.82% for Cu^2+^ in 10 repeated measurements of a solution containing 50 μg/L of both ions (Figure S2). Some reported electrochemical sensors for the simultaneous determination of Pb^2+^ and Cu^2+^ were listed in Table 1. The electrode type, electrode modification materials, linear range, and limit of detection for Pb^2+^ and Cu^2+^ were compared. Our method demonstrated competitiveness in a comprehensive consideration of linear range and limit of detection. In addition, we also compared our work with some instrumental analysis methods, such as spectrophotometry and atomic absorption spectroscopy (Table 1). It can be seen that our method has comparable sensitivity under the premise of rapid analysis and low cost.

In addition, the influence of different metal ions on the responses of Pb^2+^ and Cu^2+^ were investigated. Ca^2+^, Mg^2+^, Fe^3+^, Na^+^, and K^+^ with a concentration ten times that of Pb^2+^ and Cu^2+^ were added to the test solution as interfering ions. As shown in Figure 6d, when the interfering ion exists, there are no obvious changes in the stripping peak current of both Pb^2+^ and Cu^2+^, indicating that Ca^2+^, Mg^2+^, Fe^2+^, Na^+^, and K^+^ have little interference on the determination of Pb^2+^ and Cu^2+^. Therefore, NPG/SPCE has great specificity and anti-interference ability.

### 3.6. Recovery Study

To assess the feasibility of NPG/SPCEs in practical applications, a spike-recovery method was employed to detect Pb^2+^ and Cu^2+^ in bottled drinking water and tap water samples. The spiking amounts of Pb^2+^ and Cu^2+^ were set to four different levels. The water sample was pretreated following the guidelines in Section 2.4 and each spiked sample was tested three times in parallel. Then the recovery rates and RSD were calculated. To further validate the detection accuracy of the constructed electrochemical sensor, the spiked water samples were also tested using ICP-MS for comparison.

As shown in Table 2, when NPG/SPCE was used to detect bottled water and tap water samples, the recovery rates of Pb^2+^ ranged from 94.5% to 102.8%, with an RSD of 1.01% to 3.82%, and the recovery rates of Cu^2+^ were between 98.1% and 107.5%, with an RSD of 1.04% to 3.68%. In addition, the determination values were basically consistent with the ICP-MS detection results with a minimal deviation less than 5%, indicating that the heavy metal sensor has good accuracy and reliability.

## 4. Conclusions

In this study, a nanoporous gold film was electrodeposited onto the surface of SPCE using the hydrogen bubble template method. The electrodeposition potential and electrodeposition time were optimized according to the active surface area of prepared NPG/SPCEs. The nanoporous gold demonstrated superior electron transfer ability due to its 3D nanostructure. The NPG/SPCE was used to construct an electrochemical sensor for the simultaneous detection of Pb^2+^ and Cu^2+^. With its broad linear dynamic range, low detection limit, and high recovery rates in real sample analysis, this electrode demonstrates suitability for quantitative determination of Pb^2+^ and Cu^2+^ in drinking water. Overall, this sensor offers simplicity in terms of electrode modification and detection process, making it highly promising for lead and copper determination applications in environmental monitoring and food safety.

## Figures and Tables

**Table 1:** Comparison of different electrochemical sensors and instrumental analysis methods for the simultaneous determination of Pb^2+^ and Cu^2+^.

Electrode Substrate	Electrode Modification	Linear Range (μg/L)		Limit of Detection		Applications	References
		Pb^2+^	Cu^2+^	Pb^2+^	Cu^2+^		
Screen-printed gold electrode	GNP ^1^	20–200	20–200	2.2	1.6	-	[36]
Glassy carbon electrode	Ce-CNFs ^2^	0.9–2.1	0.6–1.8	0.6	0.3	River water	[37]
SPCE	Calixarene	100–2400	100–2400	38	40	Waste water	[38]
Carbon paste electrode	RGO/Bi ^3^	20–120	20–100	0.55	26	Ground-water	[39]
Atomic Absorption Spectrometry (AAS)		250–1500	250–1500	28.4	3.7	Fish	[40]
Inductively Coupled Plasma Optical Emission Spectrometry (ICP-OES)		/	/	0.4	0.5	Seawater	[41]
Laser-Induced Breakdown Spectroscopy (LIBS)		/	/	125	12	water	[42]
SPCE	NPG	1–100	10–100	0.4	5.4	Tap-water	This work

^1^ GNP denotes gold nanoparticles; ^2^ Ce-CNFs are cerium oxide-catalyzed carbon nanofibers; ^3^ RGO/Bi is a nanocomposite of reduced graphene oxide and bismuth.

**Table 2:** Detection results of spiked samples measured by the electrochemical method and ICP-MS.

Sample	Spiked (μg/L)		Found (μg/L)		Recovery Rate (%)		RSD * (%)		Found by ICP-MS (μg/L)		|Error| (%)	
	Pb^2+^	Cu^2+^	Pb^2+^	Cu^2+^	Pb^2+^	Cu^2+^	Pb^2+^	Cu^2+^	Pb^2+^	Cu^2+^	Pb^2+^	Cu^2+^
Bottled water	0		-	-	-	-	-	-	0.18	0.46	-	-
	30		29.2	29.6	97.4	98.5	1.01	1.04	29.6	30.9	1.35	4.21
	60		56.7	58.9	94.5	98.1	2.78	2.12	61.2	58.8	7.35	0.17
Tap water	0		-	-	-	-	-	-	0.32	1.32	-	-
	30		30.8	32.3	102.8	107.5	2.90	3.20	31.0	31.7	0.65	1.89
	60		60.2	63.3	100.3	105.5	3.82	3.68	60.5	61.9	0.50	2.26

* Relative standard deviation. For each concentration, three replicates were measured.

## Data Availability

All data are contained within this article or the Supplementary Materials.

## Supplementary Materials

The following supporting information can be downloaded at: https://www.mdpi.com/article/10.3390/s24175745/s1, Figure S1: The detection results of 10 repeated tests for the individual determination of Pb^2+^ (a) and Cu^2+^ (b) with a concentration of 50 μg/L; Figure S2: The detection results of 10 repeated tests for the simultaneous determination of Pb^2+^ and Cu^2+^ with a concentration of 50 μg/L each.
