# Supplementary material for: Nanoporous Gold-Modified Screen-Printed Electrodes for the Simultaneous Determination of Pb2+ and Cu2+ in Water
Source: Sensors (Basel). 2024 Sep 4;24(17):5745. doi: 10.3390/s24175745 (PMC11397832; doi:10.3390/s24175745)
Supplement: Supplementary file 1 [file sensors-24-05745-s001.zip › sensors-3178421-supplementary.pdf]

Supplementary Materials

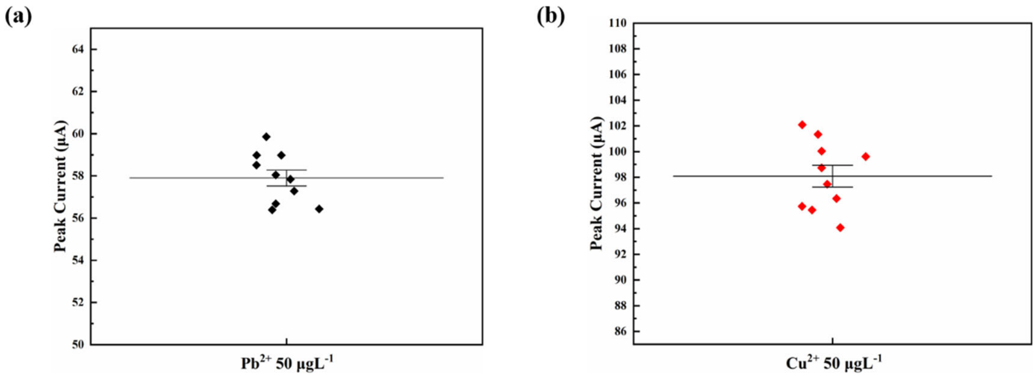

Figure S1. The detection results of 10 repeated tests for the individual determination of Pb^{2+} (a) and Cu^{2+} (b) with a concentration of 50 μg/L.

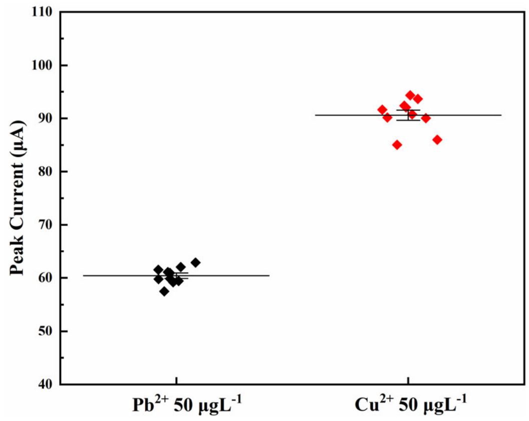

Figure S2. The detection results of 10 repeated tests for the simultaneous determination of Pb^{2+} and Cu^{2+} with a concentration of 50 μg/L each.
